# Evaluation of Microleakage and Micromorphological Analysis of Different Self-Adhesive Restorative Systems in Class V Cavities: Laboratory Study 

**DOI:** 10.4317/jced.62843

**Published:** 2025-07-01

**Authors:** Mohamed E. Hamouda, Youniss S. Harp, Abeer E. Elembaby

**Affiliations:** 1 Assistant lecturer, BDS, MDS, Department of Conservative Dentistry, Faculty of Dentistry, Mansoura University, Mansoura, Egypt, Algomhoria Street, Mansoura, Aldakhlia, Egypt P.O; 2 Lecturer, BDS, MDS, PhD, Department of Conservative Dentistry, Faculty of Dentistry, Mansoura University, Mansoura, Egypt, Algomhoria Street, Mansoura, Aldakhlia, Egypt P.O; 3 Professor, BDS, MDS, PhD, Department of Conservative Dentistry, Faculty of Dentistry, Mansoura University, Mansoura, Egypt, Algomhoria Street, Mansoura, Aldakhlia, Egypt P.O

## Abstract

**Background:**

Microleakage is a common problem that affects the longevity of restorative materials in Class V cavities. It is influenced by factors such as the type of restorative materials, composition of the cavity margins and aging. This study aimed to evaluate and compare microleakage and micromorphological analysis of restoration-dentin interface for Class V cavities restored with resin modified glass ionomer cement, self-adhesive flowable composite and self-adhesive composite hybrid before and after thermocycling for 10000 cycles.

**Material and Methods:**

One hundred two sound premolars received standardized Class V cavities on their buccal surfaces with the gingival margin placed 1mm above the cementum-enamel junction. The prepared premolars were randomly divided into three groups according to the self-adhesive restorative systems used (n=34). Each group was further subdivided into two subgroups according to aging (n=17); the first one was immediately evaluated after 24 hours of restoration placement, while the second was evaluated after thermocycling. Restored premolars were evaluated using dye penetration microleakage test under a stereomicroscope and micromorphological analysis of restorations/dentin interface. Microleakage scores were statistically analyzed using Kruskal-Wallis test, Mann-Whitney U test and Wilcoxon Signed Rank test.

**Results:**

There was a statistically significant difference regarding microleakage between the used self-adhesive restorative systems (*p*<0.05). Self-adhesive composite hybrid had the highest microleakage scores followed by self-adhesive flowable composite while resin modified glass ionomer cement had the lowest microleakage scores. Also, there was a significant increase in the microleakage scores for the used restorative systems after thermocycling (*p*<0.05).

**Conclusions:**

Resin modified glass ionomer cement remains the material of choice for Class V cavities in the term of microleakage compared to self-adhesive flowable composite and self-adhesive composite hybrid. The sealing stability of the used self-adhesive restorative systems still questionable over time.

** Key words:**Class V cavities, microleakage, resin modified glass ionomer cement, self-adhesive composite, thermocycling.

## Introduction

The longevity of restorations in Class V cavities is mainly based on a good marginal adaptation and sealing ability with enamel and dentin. Class V cavities have a unique design since their margins are partially placed on enamel and partially on dentin/cementum (1,2). This variability affects the bonding quality of restorative materials with the different tooth substrate (1,3). Furthermore, they have high C-Factor as the restorations are bonded to five surfaces (3,4). This property increases the negative effects of volumetric changes for polymerizable restorative materials (4). Microleakage is considered a major cause for restoration failure in Class V cavities (3). It is defined as the passage of fluids and bacteria between restorative materials and cavity walls (1,5). It results in post-operative sensitivity, secondary caries, deterioration of adhesion and debonding of restorative materials (1,3,6).

Resin composite became the preferred restorative material for many patients as it is a tooth-colored restoration. However, it had some drawbacks as polymerization volumetric shrinkage that disrupted the internal adaptation and increased the interfacial gaps between the restoration and the cavity walls. Incremental placement technique was developed to overcome polymerization shrinkage. However, it was time consuming and increased the incidence of voids incorporation between layers (7). Another drawback of resin composite was the high stiffness due to its high filler content (8). This made it unable to compensate for contraction stress during polymerization and resulted in bond failure or fracture of the tooth structure (8) .Another drawback was the mismatch of linear coefficient of thermal expansion (LCTE) between resin composite and tooth substrate that increased the risk of marginal gaps on the exposure to the intraoral thermal stresses (7). The key of success for a restorative material in Class V cavities is to have a similar modulus of elasticity, coefficient of thermal expansion to tooth structure and good sealing ability (9).

Self-adhesive materials were considered major developments for direct restorative materials because of the absence of a specific adhesive protocol and easy manipulation (10). Also, bulk-fill technique simplified procedures by limiting the number of increments needed to fill an entire cavity (7,10). Self-cured glass ionomer cement (GIC) exhibited chemical adhesion to the tooth structure in addition to its biocompatibility (5,11,12). Furthermore, it had a similar coefficient of thermal expansion to enamel and dentin (8,13). This made it suiTable as a restorative material in Class V cavities (8). However, it had inferior mechanical properties and esthetics when compared with resin composite (5,11).

Many trials were made in order to improve the mechanical properties of conventional GIC; one of them was incorporation of urethane dimethacrylate (UDMA) monomers to develop resin modified glass ionomer cement (RMGIC) that was introduced in the late 1980s (1,11,12) This improved the mechanical properties, esthetics and increased the resistance to early moisture contact and desiccation (5,11). In addition to chemical adhesion, it also mechanically interlocked with dentin.(1,12) The final RMGIC set restoration contained an amount of resin between 4.5% to 6%. This resin component may affect the marginal seal against the tooth substrate and the development of the ionic crosslink (12).

Another trial to overcome the high stiffness of packable resin composite was the development of a flowable composite with low filler content in 1996 (13). This modification allowed flowable composite to flow and compensate the volumetric polymerization shrinkage before reaching the gel point (13,14). Recently, self-adhesive flowable composite (SAFC) was developed in order to simplify Class V cavities restorative procedure (9,15-17). This approach combined self-adhesive monomers such as 4-methacryloxyethyl trimellitic acid (4-META) or 10 methacryloyloxydecyl dihydrogen phosphate (10-MDP) with flowable composite (9,15-17). That restorative system saved time as no more need for separate etching, bonding and restoration placement steps. It depended on a dual-adhesion mechanism; chemical interaction of functional monomers with hydroxyapatite crystals and micromechanical infiltration of the resin tags with the tooth substrate (9,15,17).

Another development of self-adhesive restorative systems was the self-adhesive composite hybrid (SACH) as Sure-Fill One (10,12,18,19). It combined resin composite with a modified high molecular weight polyacrylic acid that was functionalized with polymerizable group (MOPOS) that promoted chemical adhesion with the tooth substrate (10,12,18). Also, amide based crosslinking agent (BADEP) was added in its composition to crosslink the functionalized polyacrylic acid chains together and improve mechanical strength (10,12,18) That restorative system had a dual-cure mechanism; acid–base reaction and photo polymerization reaction (10,12,18).

Self-adhesive restorative systems had a higher viscosity than the conventional liquid dental adhesives. This could interfere with their wetting ability to the dental hard tissues and adhesion performance (17). The evidence to use self-adhesive restorative systems instead of conventional resin composite in Class V cavities is sparse and more laboratory tests are needed to evaluate their long term performance. According to the mentioned before and the availability of different self-adhesive restorative systems, more studies are needed to help clinicians for a proper restorative material selection for Class V cavities. The null-hypotheses of the current study were that there would not be significant differences between RMGIC, SAFC and SACH in the term of microleakage and micromorphological analysis of restoration/dentin interface before and after thermocycling.

## Material and Methods

- Materials:

Materials used in the current study were RMGIC (Fuji II LC, GC, Tokyo, Japan), SAFC (Fusio Liquid Dentin, Pentron Clinical 1717 West Collins Orange, CA, USA) and SACH (Surefil One, Dentsply-Sirona, Konstanz, Germany). The used materials were fully described in  Table 1.

- Methods:

Sample Size Calculation

Sample size calculation was based on the results of different self-adhesive restorative materials retrieved from a previous research.(20) G power program version 3.1.9.7 was used to calculate the sample size based on an effect size of 0.36, using 2-tailed test, α error = 0.05 and power = 90.0% . The total calculated sample size was 102 (divided into 3 groups; each 34).

Ethical Approval and Teeth Selection 

Ethical approval for the current study was obtained from the ethics committee in the Faculty of Dentistry, Mansoura University under a protocol number (A0103023CD). A Total number of 102 freshly extracted sound premolars were used in this study. The teeth were collected from oral and maxillofacial surgery clinic in the Faculty of Dentistry, Mansoura University. They were extracted from patients in the age from 50 to 60 years due to periodontal diseases. Previously restored, visibly cracked, fractured, deformed, carious and abrased teeth were excluded from the study. Informed consents were obtained from the patients to use their extracted premolars for a research purpose. The teeth were cleaned from any remaining attached soft tissues and hard deposits using a hand scaler (Zeffiro; Lascod, Florence, Italy). After that, the teeth were disinfected by immersion in 0.5% chloramine at 4°C and stored in distilled water in an incubator (BTC, Model: BT1020, Cairo, Egypt) at 37°C until used (21). The extracted teeth were used within three months from extraction.

Teeth Mounting and Preparation Procedures

Polyvinyl chloride cylinders were used as molds for the production of self-cured acrylic resin blocks (Acrostone, Egypt) into which teeth were fixed so that acrylic block is beyond cementum/enamel junction by 1 mm. Standardized Class V cavities (2mm depth, 3mm occluso-gingival length and 4mm mesio-distal width) were prepared on the buccal surface of the premolars using carbide fissure bur No. 271 (Komet-Brasseler, Lemgo, Germany) at high speed with air/water coolant (21). A new bur was used for each of five preparations and then replaced (21). The gingival margins of cavities were placed in the enamel 1mm above the cementum-enamel junction with the help of a template (metallic tofflemire band with 2D window design with the cavity dimensions) for a uniformly shaped outline for all preparations (22). Cavity depth was standardized by placing a rubber stopper at the level of 2mm at the bur working part and assessed using a graduated periodontal probe (Zeffiro; Lascod, Florence, Italy). All cavo-surface angles were kept at 90 degrees without any bevel designs.

Restorative Procedures

The prepared premolars were randomly divided into three groups according to the self-adhesive restorative systems used (n=34); Group R1 was restored with RMGIC (Fuji II LC), Group R2 was restored with SAFC (Fusio Liquid Dentin) and Group R3 was restored with SACH (Surefil One). Cavities were restored with RMGIC, SAFC and SACH according to their manufacturer’s instructions. Restorative procedures were done by a single operator to decrease variability. Prophylaxis with a non-fluoridated polishing paste (Prophy Paste, Kent, UK), prophy brush and water was performed for all cavities before the restorative procedures. Dentin conditioning was done for RMGIC group using aqueous solution of 10% poly-acrylic acid (Dentin Conditioner Liquid, GC, Tokyo, Japan) for 20 seconds. After that, cavities were rinsed well and dried with cotton pellets to avoid desiccation (23).

RMGIC and SACH capsules were mixed using an amalgamator (Linea Tac 200/s, Serravalle, Italy) for 10 seconds and immediately evacuated into cavities without any primer application. Both restorative materials were adapted to the cavities using gold-plated dental composite applicator (Sedra Dent, Cairo, Egypt). Further adaptation was done with the aid of transparent celluloid strips (TOR VM Ltd, Moscow, Russia) that were wrapped all around the teeth crowns. After that, both restorative materials were light cured for 20 seconds using a light emitting diode (LED) light curing unit with light intensity of 1200 mw/cm2 (Bluephase 20i, Ivoclar Vivadent, Schaan, Liechtenstein). The light curing distance was standardized using an orange light shield adjusted to bring light curing tip 1mm away from the restorative materials.

SAFC was directly applied to cavities without any conditioning or adhesive application. It was applied into cavities in the form of two increments. The first increment of 1mm was applied into cavities with agitation motion against enamel and dentin for 20 seconds using the syringe tip then light cured for 10 seconds. That thickness was adjusted by placing a notch at 1mm height of the syringe tip. The second increment was then applied and light cured for 10 seconds. Light intensity of the used LED was regularly assessed using a dental radiometer (Bluephase Meter, Ivoclar Vivadent AG, Schaan, Liechtenstein). Restorations were finished using tungsten carbide bur (Okodent, Tautenhain, Germany) and polished using medium, fine and extra-fine grits diamond Sof-Lex™ discs (3M ESPE, St. Paul, MN, USA). The restorations/teeth interfaces were inspected for any excessive restorative materials overlap using magnifying loupes (4 Loupes, Amtech, Wenzhou, China) under magnification of 4X and LED head light (HLP05, Amtech) illumination. A thin layer of nano-filled self-adhesive protective coat (G-Coat Plus, GC, Tokyo, Japan) was applied using a micro-brush and light-cured for 20 seconds on the outer surface of RMGIC restorations (21).

Thermal Cycling

**Figure 1:**
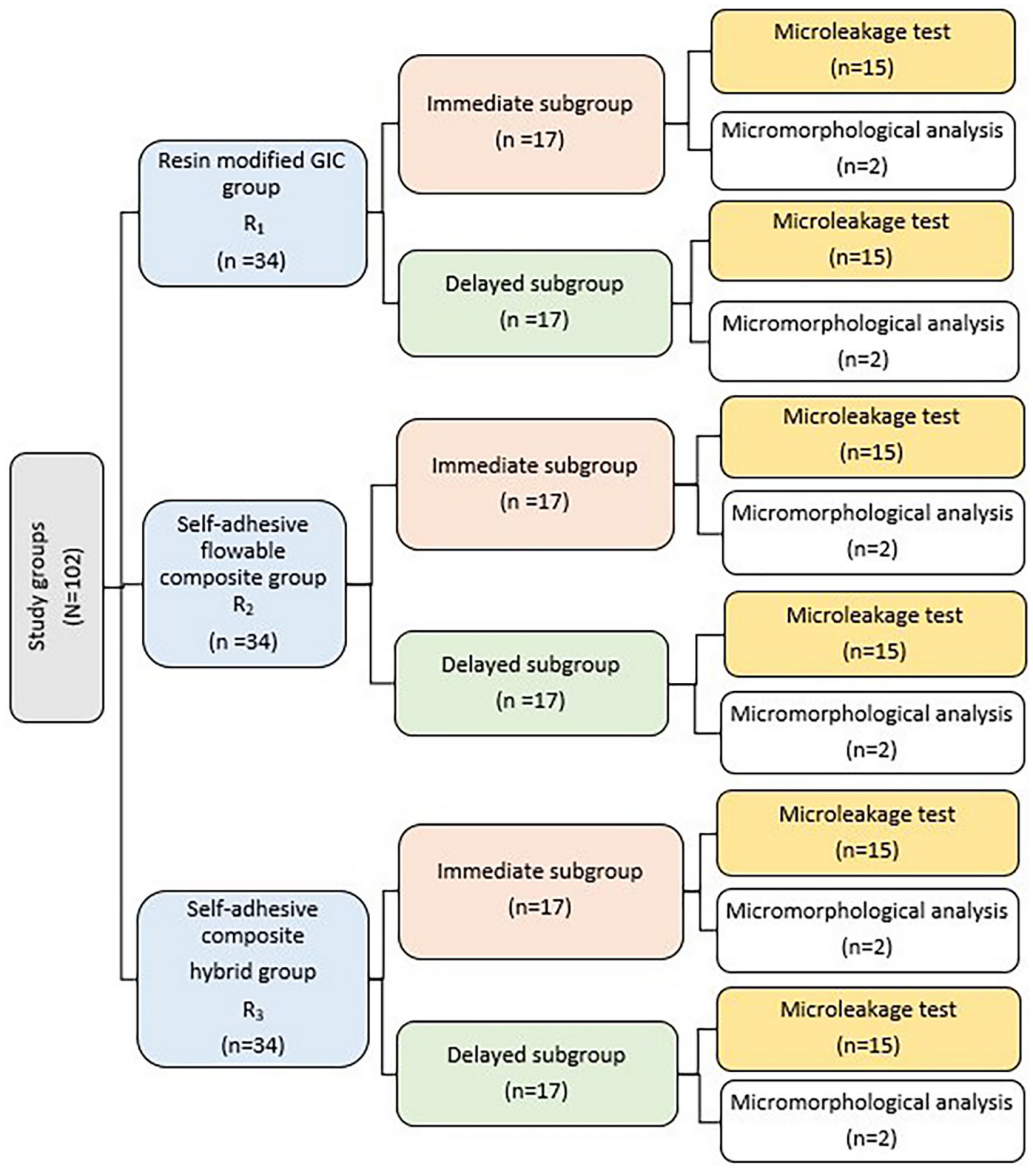
Flow chart showing the study design.

Half of the specimens from each group (n=17) was evaluated immediately after 24 hours of restorations placement. A number of 15 specimens were evaluated using dye penetration microleakage test and the remaining 2 specimens were evaluated for micromorphological analysis of restoration/dentin interface. The other half was thermocycled (SD Mechatroniks thermocycler, Germany) for 10000 cycles between water paths held at 5°C and 55°C with a dwell time of 30 seconds in each bath. After that, it was evaluated as the immediate subgroups as shown in Figure 1.

Microleakage Assessment 

**Figure 2:**
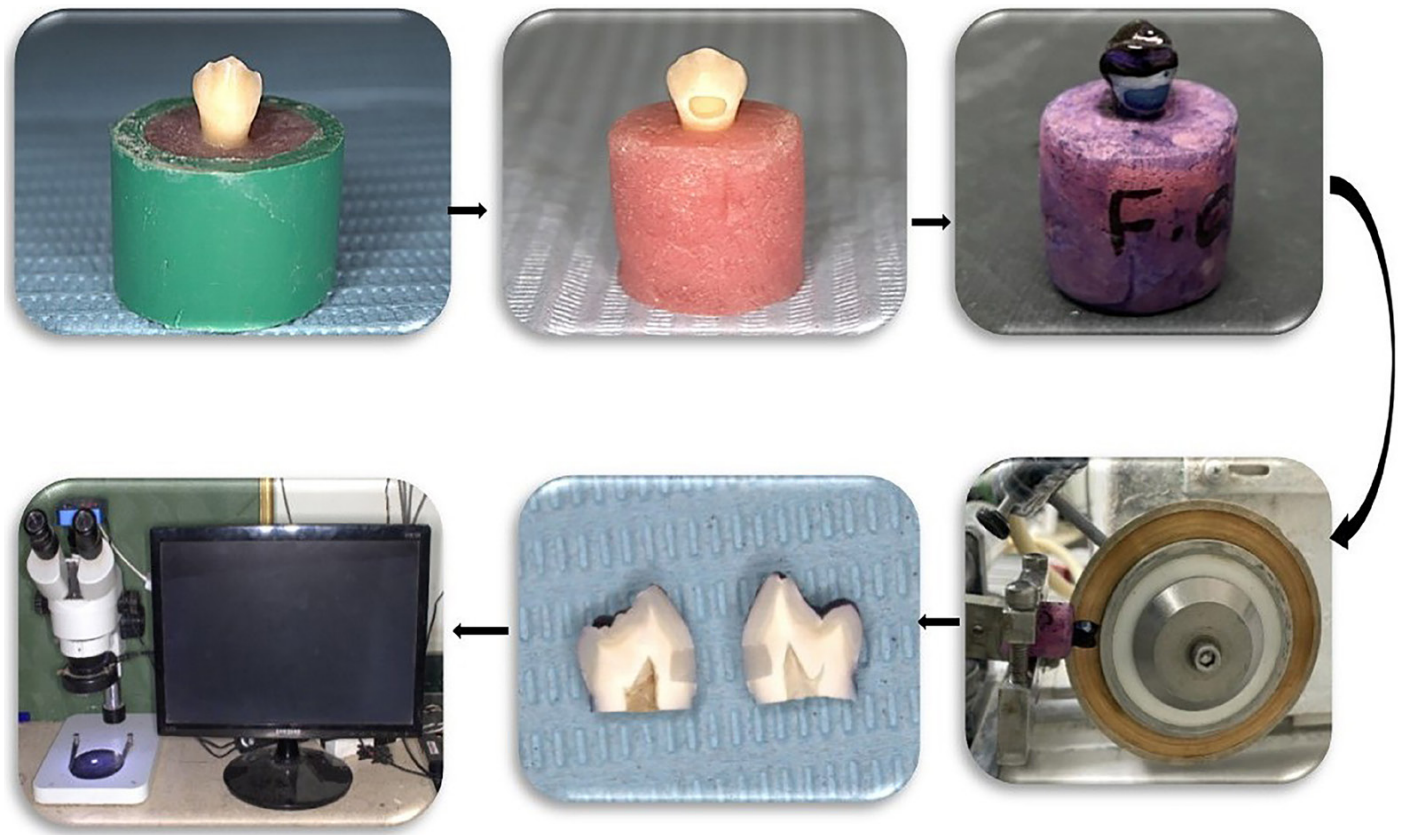
Specimen preparation for dye penetration microleakage evaluation.

The surfaces of the specimens were covered with two layers of nail varnish then; each tooth was sealed with a layer of sticky wax except for the restorations and 1 mm rim of the tooth structure around them (21,23,24). The restored teeth were immersed in 2% methylene blue dye for 24 hours at the room temperature. Then, they were removed from the solution, rinsed with distilled water for 30 seconds (21,23,24). Each tooth was sectioned bucco-lingually through the center of the restoration using a water-cooled slow-speed diamond micro saw Isomet 4000 (Buehler, Ltd, Lake Bluff, LL, USA) with 0.3-mm thick diamond coated disc at 2050 rpm; 8.8 mm/min feeding rate (21,24). After that, a final horizontal cut was done 1mm below cementum/enamel junction in order to separate the sections. The sections were examined using an optical stereomicroscope at 40X (MA 100 Nikon stereomicroscope, Japan) to determine the extension of the dye penetration at both occlusal and gingival margins as shown in Figure 2 (21). Dye penetration was evaluated by a single operator to decrease variability and scored according to the following scale (9,24).

0. Absence of marginal dye penetration.

1. Presence of dye penetration but not exceeding the middle of the cavity depth.

2. Presence of dye penetration past the middle of the cavity depth.

3. Dye penetration along the axial wall.

Micromorphological Analysis

The restored specimens were sectioned bucco-lingually in two equal halves along the long axis of teeth using the Isomet. Each half was polished with 600-grit, 1000-grit, 1200-grit, 2000- and 4000-grit silicon carbide paper respectively. After that, specimens were cleaned using ultrasonic cleaner (CD-4820, China) for 10 minutes to get rid of any debris. Then, specimens were placed in 10% orthophosphoric acid for 10 seconds and rinsed with distilled water for 10 seconds. Furthermore, they were placed in 5% sodium hypochlorite for 5 minutes then rinsed with distilled water for 10 seconds. Specimens were gold sputtered twice (SPI Module -Sputter Carbon / Gold Coater, EDEN instruments, Japan) and observed at a secondary electron detection mode under a scanning electron microscopy (JSM-6510LV, JEOL, Japan) at an accelerating voltage of 20 KV and at a working distance of 9-13 mm. Specimens were imaged at different levels of magnification (1000x, 2000x) to describe the micromorphology of restorations/dentin interface.

## Results

The dye penetration microleakage test scores were tabulated at an excel sheet and statistically analyzed using SPSS for Windows statistical software (version 12.0.1; SPSS Inc., Chicago, IL, USA). They were analyzed using Kruskal-Wallis test to check any significant differences among the different self-adhesive restorative systems used. Mann-Whitney U test and Wilcoxon signed rank test were used for pair wise comparisons. Significance was evaluated at *P* < 0.05 for all tests.

Microleakage Test Results

**Figure 3:**
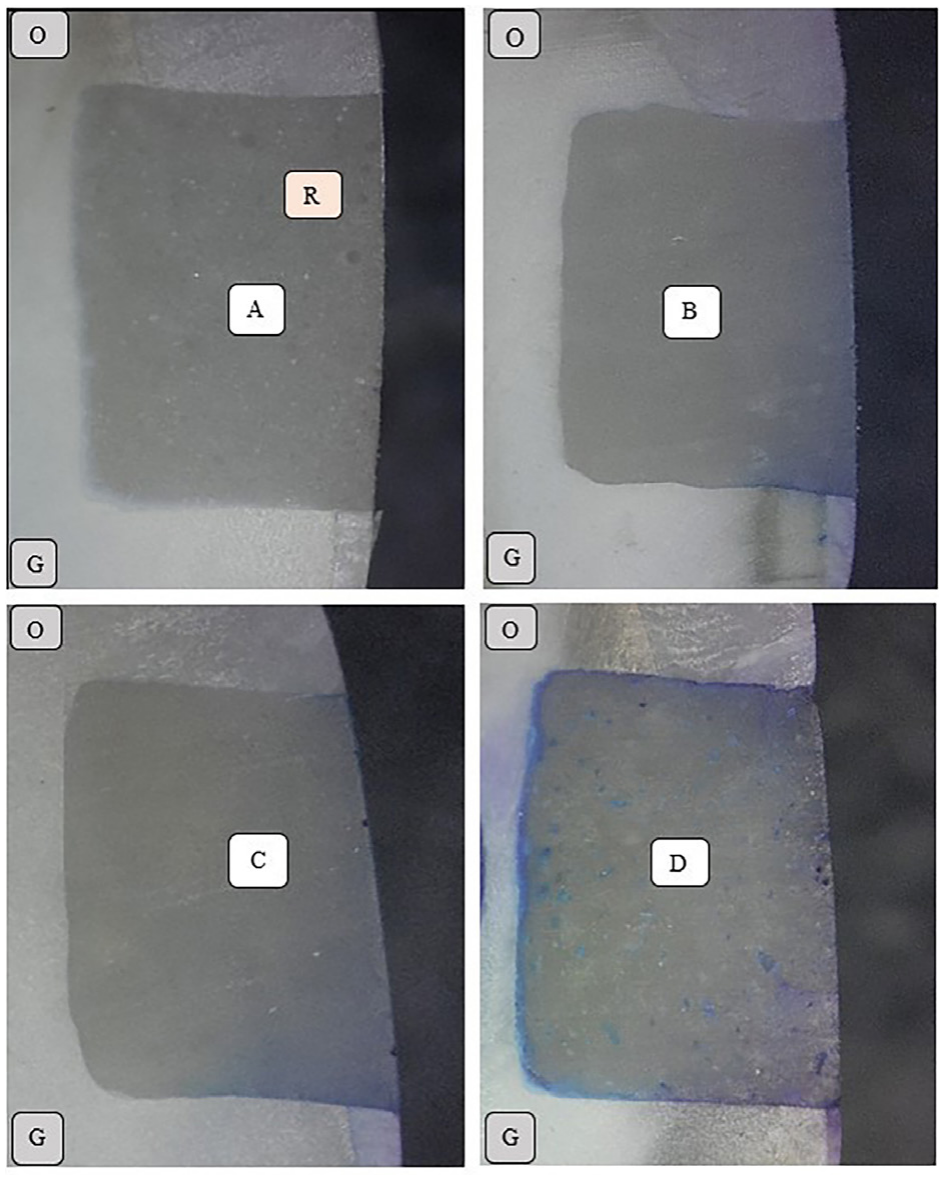
Microleakage scores evaluation. (A) Score 0, (B) Score 1, (C) Score 2, (D) Score 3. O, Occlusal; G, Gingival; R, Restoration.

Kruskal-Wallis test revealed a statistically significant difference for immediate and delayed subgroups at both the occlusal and the gingival margins (*p*<0.05). Among immediate subgroups, Mann-Whitney U test revealed that SACH had the highest microleakage scores among immediate subgroups at the gingival margin followed with SAFC while RMGIC had the lowest microleakage scores. Wilcoxon Signed Rank test revealed that each immediate subgroup had no statistically significant difference for microleakage scores between its occlusal and gingival margins (*p*>0.05). For delayed SACH subgroup, Mann-Whitney U test revealed a significant increase in microleakage at gingival margin (*p*=0.007) while there was no significant increase in microleakage at occlusal margin (*p*=0.200). For delayed SAFC subgroup, Mann-Whitney U test revealed a significant increase in microleakage at both the gingival margins (*p*=0.008) and the occlusal margins (*p*=0.020). Wilcoxon Signed Rank test revealed a statistically significant difference between gingival and occlusal margins for delayed SAFC subgroup (*p*=0.040). For delayed RMGIC subgroup, Mann-Whitney U test revealed a significant increase in microleakage at the occlusal margins (*p*=0.00) while there was no significant increase in microleakage at the gingival margins (*p*=0.102). Wilcoxon Signed Rank test revealed a statistically significant difference between gingival and occlusal margins for delayed RMGIC subgroup (*p*=0.017). Microleakage results were presented in  Table 2, Table 3, Table 4. Representative photographs of the dye penetration microleakage were presented in Figure 3.

Micromorphological Analysis Results

**Figure 4:**
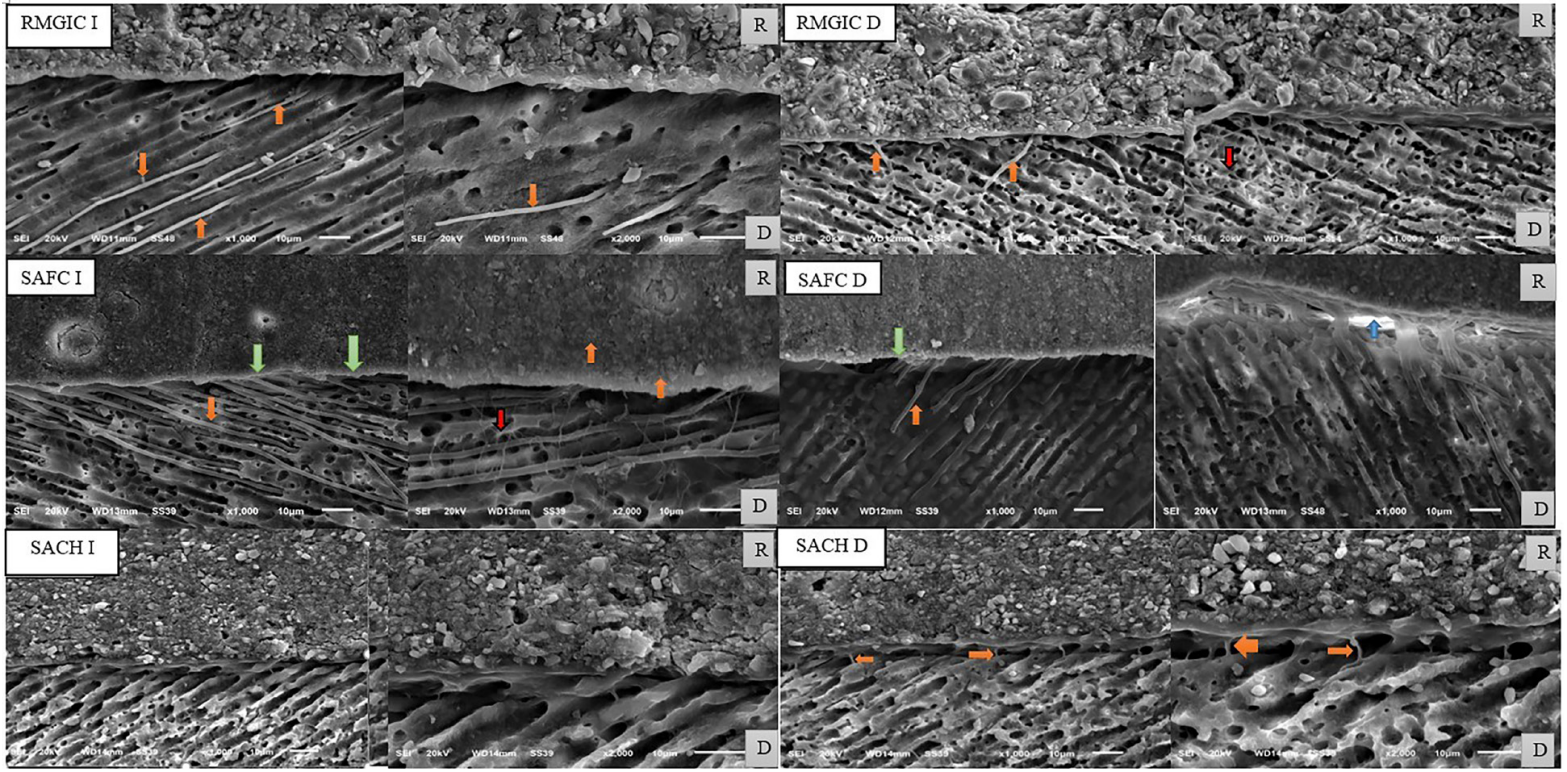
SEM image showing the micromorphology of restoration/dentin interface for different study subgroups; green arrows represent hybrid layer, orange arrows represent resin tags, red arrows represent resinous meshwork, blue arrow represents interfacial gap, D represents dentin and R represents restoration.

For immediate RMGIC subgroup a relatively intimate adaptation with no clear formation of hybrid layer at the restoration-dentin interface were illustrated. Long cylindrical resin tags with funnel origin configuration were noticed inside dentinal tubules (122:132 μm in length). For delayed RMGIC subgroup short cylindrical resin tags were noticed inside dentinal tubules (4.5:25.5 μm in length). Also, a resinous network extending into the dentinal tubules and lateral canaliculi was illustrated. A small budding conFiguration of resin tags was noticed as shown in Figure 4.

For immediate SAFC subgroup a thin hybrid layer along with signs of long thick cylindrical resin tags infiltration were noticeable (130:143 μm in length). A thin resinous meshwork extending through lateral dentinal canaliculi was noticed bridging intra-tubular resin tags together. For delayed SAFC subgroup a thin hybrid layer along with signs of short cylindrical resin tags infiltration were noticeable (5:72 μm in length). Resin tags were teared and non-continuous when compared with immediate SAFC subgroup. Also, the resinous meshwork that was showed in the immediate SAFC subgroup disappeared. Furthermore, restoration/dentin interfacial gaps (5:9 μm) were demonstrated as shown in Figure 4.

For immediate SACH subgroup a relatively intimate adaptation with no clear formation of hybrid layer at the restoration-dentin interfaces was illustrated. No signs for resin tags infiltration into dentinal tubules were observed. For delayed SACH subgroup absence of acid-base resistant layer (resembling voids) and very thin short resin tags were observed at the restoration/dentin interface (2:5 μm in length) as shown in Figure 4.

## Discussion

Class V cavities were used in the current study to evaluate self-adhesive restorative systems for multiple reasons. First, they represent restorative challenges for the different types of restorative materials as they have a complex morphology because the cavities occlusal margins have high enamel/dentin ratio than the gingival margins (17). Second, they represent high stress clinical situations owing to their high C-Factor as restorations are bonded to five surfaces that represent challenges for the different adhesive restorative systems (17). They increase the shrinkage stresses at the adhesive interface and decrease the sealing ability (17). Third, Class V cavities restorative procedures are simple and minimal consequently lowering the operator variability (17). Fourth, both occlusal and gingival margins can be evaluated in these types of cavities (3,17). Finally, they have many challenges during isolation and continuously exposed to high mechanical micro-shear stresses during mastication (3,24). These factors made them suiTable for the evaluation of the different self-adhesive restorative materials.

Microleakage at restoration/tooth interface is considered one of the major factors that affects the longevity of the restoration (6,14). It acts as a seed sower for the development of secondary caries, teeth sensitivity and pulp inflammation (3). Furthermore, it allows oral fluids and bacteria to invade the restoration/tooth interface resulting in deterioration of the bonded area and adhesive failure (11,24). Marginal seal can be affected by many factors as bonding to tooth structure, polymerization shrinkage of the restorative materials, elastic modulus and linear coefficient of thermal expansion of the restorative material and the tooth structure (9,23). Dye penetration microleakage test was used in the current study to evaluate the performance of different self-adhesive restorative materials. It is a valid, precise, cheap, fast, easy to perform and accurate method for microleakage detection with 82% accuracy (25). The 2% methylene blue dye is simple to use (21,25). Also, it has high water solubility and rapid diffusion into imperfections and cracks in the tooth substrate as it has a smaller molecular size than typical bacteria with a diameter of 0.80 nm (4,22). Furthermore, it has low molecular weight, no color changes during test and lack of high transmission of incident light (21,22). Moreover, it has no tendency for bonding to the tooth structure or restorative materials (4).

Thermocycling was considered the preferred method of aging (15,23,26). Half of current study specimens were subjected to aging. In order to simulate the different thermal changes in the oral cavity; specimens were thermocycled for 10000 cycles between 5°Cand 55°C with a dwell time of 30 seconds to create thermal stresses at the adhesive joint at the tooth/restoration interface (9,17). This highlighted the mismatch in linear coefficient of thermal expansion between the tooth structure and the restorations (9,17,21). Also, it resulted in different volumetric changes during the different thermal alterations (11,17,21). These stresses resulted in thermal cyclic fatigue for the adhesive joint, degradation of bond and increased the incidence of microleakage (9,17).

 Microleakage results of the current study were affected with the type of restorative materials, the location of cavity margin and aging. Immediate RMGIC subgroup showed the lowest microleakage test scores among the study subgroups. It also revealed no significant difference between occlusal and gingival margins. This could be explained with its low modulus of elasticity that relieved the developed stresses from the restorative material polymerization shrinkage (13,23). This property enabled RMGIC to have a higher capacity for plastic flow and stress relaxation during polymerization (13,23). Also, the two-fold adhesion mechanism enabled RMGIC to bond both chemically and mechanically (hybridization) to enamel and dentin and improved the adhesion to the tooth substrate (1,13,14). Furthermore, pretreatment of the cavities with poly-acrylic acid improved the bond effectiveness (21). Moreover, application of nano-filled protective coat filled the marginal gaps between the restorations and cavity walls, improved the marginal sealing and decreased the incidence of microleakage (21,27). A previous study agreed with these findings (21).

Delayed RMGIC subgroup showed significant increase in dye penetration scores at the occlusal margins without increase in dye penetration at the gingival margins. This could be returned to the mismatch of linear coefficient of thermal expansion between RMGIC (25.4:30 ppm) and the tooth substrate; enamel (17 ppm) and dentin (11 ppm) (23). Different cycles of expansion and contraction with the mismatch of LCTE between restoration and cavity margins increased the incidence of debonding and microleakage (23). These results were supported with the findings of a previous study and could be explained with RMGIC adhesion mechanism (28). Adhesion of RMGIC depended mainly on a physico-chemical reaction with enamel and dentin due to the polar nature of the polyacrylates and minerals in the dental hard tissues (28). RMGIC had a higher bond strength to dentin (27.7 MP) than to enamel (15.8 MP) (29). This justified the marginal sealing stability at the cavities gingival margins with higher dentin/enamel ratio than the occlusal margins for delayed RMGIC subgroup. The hydrophilic components (resin matrix HEMA and polyacrylic salt network) of RMGIC had water sorption tendency that resulted in a hygroscopic expansion that reversed the tensile or pulling stresses into compressive stresses (23,28). This compensatory mechanism minimized the gaps between the cavity walls and the restorations and decreased the microleakage scores (23,28).

Immediate SAFC subgroup had significantly higher microleakage scores than RMGIC. SAFC had poor bonding effectiveness in non-retentive cavities and cavities without macro-retentive means (17). This was attributed to the insufficient removal of the smear layer (15,17). SAFC had mild acidity with low etching ability that superficially interacted with the tooth structure (15). Furthermore, it had higher viscosity and less wettability than the conventional adhesive solutions leading to inadequate hybridization of the collagen mesh (15). SAFC had equal bond strength values with enamel and dentin (30). That justified the non-significant difference between occlusal and gingival margins for SAFC immediate subgroup.

Delayed SAFC subgroup revealed significant increase for microleakage scores at both occlusal and gingival margins. Previous studies evaluated immediate and aged bond strength of SAFC to the tooth substrate and revealed adhesion instability with enamel and dentin (15,31). SAFC contained hydrophilic acidic functional monomers that absorbed water overtime resulting in plasticization, debonding of fillers away from the resin monomers, filler loss, adhesive failure and increased the microleakage incidence (15,31). These findings were supported with the micromorphological analysis results of the current study that revealed degradation of resin tags and the development of interfacial gaps for SAFC delayed subgroup. A previous study reported that SAFC had more interfacial defects and gaps with dentin than enamel (32). These defects increased after aging (32). This justified the higher microleakage scores for the gingival margins (high dentin/enamel ratio) than the occlusal margins for delayed SAFC subgroup.

SACH had the highest microleakage scores in the current study. Many studies reported that bulk-fill application technique for SACH in high C-Factor cavities and its light curing according to the manufacturer instructions had negative effects on the bond strength and increased the volumetric shrinkage stresses at the restoration/ tooth interface (20,33,34). Furthermore, they increased the tendency of restoration separation from cavity walls, interfacial gaps and poor marginal adaptation (20,33). Light-initiated polymerization resulted in a short gel stage and insufficient flow to compensate the restoration contraction tensile stresses (20,33). Although SACH contained water in its composition, it required additional moisture in the tooth cavity in order to activate the functional acids to promote adhesion with the dental substrates (20,33). It was so difficult to achieve optimal wetting degree in shallow or deep cavities for SACH. Another drawback for SACH was the high viscosity that compromised the cavity surface wetting and left porosities at the restoration/tooth interface (20,33,35). On the other side, SAFC has low viscosity and better wettability and adaptation to the cavity walls than SACH. This could explain the high microleakage scores for immediate SACH subgroup when compared with immediate SAFC subgroup. Adhesion mechanism of SACH was based mainly on the chemical ionic interaction of carboxylic acid groups of MOPOS and polyacrylic acid with the calcium ions of the tooth substrate (12,18). Enamel is rich with inorganic hydroxyapatite which represents 92% of enamel volume while dentin contains only 45% inorganic hydroxyapatite volume. That justified the increase in microleakage at gingival margins (with low enamel/dentin ratio) for delayed SACH subgroup after thermocycling.

SACH had significant higher microleakage scores at both occlusal and gingival margins than RMGIC before and after thermocycling. A previous study reported that RMGIC had more bond effectiveness than SACH in high C-Factor cavities immediately after restorative procedures and after aging (30). SACH had a limited and less homogenous interfacial adaptation to dentin than RMGIC (18). These factors justified the significant difference in microleakage scores between RMGIC and SACH immediate and delayed subgroups in the current study. The study null-hypotheses were rejected as there were significant differences in dye penetration microleakage test and restorations/dentin micromorphology results between the used self-adhesive restorative systems before and after thermocycling. The current study had some limitations; one of them was that specimens weren’t exposed to a mechanical loading simulating the intra-oral masticatory forces or different PH conditions. Also, there was a lack of the natural fluid movement inside dentinal tubules as study was performed using extracted teeth.

## Conclusions

Within the limitations of the current study it was concluded that:

1. None of the self-adhesive restorative systems tested in the current study was able to totally prevent the microleakge in Class V cavities.

2. Resin modified glass ionomer cement remains the material of choice for Class V cavities in the term of microleakage than self-adhesive flowable composite and self-adhesive composite hybrid.

3. Self-adhesive flowable composite and self-adhesive composite hybrid sealing stability to the dental hard tissues still questionable and more studies are needed to evaluate their long-term performance in Class V cavities.

## Figures and Tables

**Table 1:** Materials used in the study.

Commercial name	Type and reaction	Manufacturer	Composition	Batch number
Fuji II LC	Resin modified glass ionomer cement (dual cured, self-adhesive).	GC, Tokyo, Japan.	2-Hydroxyethyl Methacrylate, polyacrylic acid, water; 58 wt.% fluoroaluminumsilicate.	220119122 2201191
Fusio Liquid Dentin	Flowable composite (light cured, self-adhesive).	Pentron Clinical 1717 West Collins Orange, CA, USA.	4-META (4-methacryloxyethyl trimellitic acid)- based flowable composite containing nano sized amorphous silica.	8751813
Surefil One	Composite hybrid (dual cured, self-adhesive).	Dentsply-Sirona, Konstanz, Germany.	powder: silanated aluminum-phosphor-strontium sodium-fluoro-silicate glass, dispersed silicon dioxide, ytterbium fluoride, pigments liquid: acrylic acid, polycarboxylic acid, bifunctional acrylate, self-cure initiator, camphorquinone, stabilizer.	2201000710
Dentin Conditioner Liquid	Dentin conditioner.	GC, Tokyo, Japan.	10% polyacrylic acid, aluminum chloride and distilled water.	2112021
G-Coat Plus	Nano-filled surface sealant (self-adhesive).	GC, Tokyo, Japan.	Urethane methacrylate, methyl methacrylate, camphorquinone, silicon dioxide, phosphoric ester monomers.	2104221

**Table 2:** Comparison of median and interquartile range of microleakage scores among the used self -adhesive restorative systems according to time and cavity margins.

		Restoration			P
		RMGIC	SAFC	SACH	
Immediate	Occlusal	.00(.00-.00)Aa	1.00(1.00-.00)Bb	2.00(2.00-1.00)Cc	.00*
	Gingival	.00(.00-.00)Aa	.00(.00-.00)Bb	2.00(3.00-1.00)Cc	.00*
Delayed	Occlusal	1.00(1.00-1.00)Ab	1.00(1.00-1.00)Ac	2.00(3.00-1.00)Cc	.004*
	Gingival	.00(.00-.00)Aa	.00(.00-.00)Bd	3.00(3.00-3.00)Cd	.00*

Abbreviations: RMGIC, resin modified glass ionomer cement; SAFC, self-adhesive flowable composite; SACH, self-adhesive composite hybrid. Note: Data expresses as median (IQR) IQR: interquartile range *: significance (*p* < 0.05). Different letters indicate to significant difference. Capital letters indicate significance among different self-adhesive restorative systems within the same aging condition at the occlusal and the gingival margins. Small letters represent significance between imme¬diate and delayed within the occlusal and the gingival margins of each restoration.

**Table 3:** Comparison of median and standard deviation of microleakage scores between occlusal and gingival cavity margins according to restoration and time.

		Immediate		P1	Delayed		P2
		occlusal	cervical		occlusal	cervical	
RMGIC	Median	.00	.00	1.00	1.00	.00	.017*
	IQR	.00-.00	.00-.00		1.00-1.00	.00-.00	
SAFC	Median	1.00	.00	.257	1.00	.00	.040*
	IQR	1.00-.00	.00-.00		1.00-1.00	.00-.00	
SACH	Median	2.00	2.00	.710	2.00	3.00	.020*
	IQR	2.00-1.00	3.00-1.00		3.00-1.00	3.00-3.00	

IQR: interquartile range P: Probability *: significance <0.05 Test used: Mann Whitney

**Table 4:** Comparison of median and standard deviation of microleakage scores between Immediate and delayed time according to restoration and cavity margins.

		occlusal		P1	gingival		P2
		Immediate	Delayed		Immediate	Delayed	
RMGIC	Median	.00	1.00	.00*	.00	.00	.102
	IQR	.00-.00	1.00-1.00		.00-.00	.00-.00	
SAFC	Median	1.00	1.00	.020*	.00	.00	.008*
	IQR	1.00-.00	1.00-1.00		.00-.00	.00-.00	
SACH	Median	2.00	2.00	.200	2.00	3.00	.007*
	IQR	2.00-1.00	3.00-1.00		3.00-1.00	3.00-3.00	

IQR: interquartile range P: Probability *: significance <0.05 Test used: Mann Whitney

## Data Availability

The datasets used and/or analyzed during the current study are available from the corresponding author upon reasonable request.
